# Heterozygous Hereditary Vitamin D-Dependent Rickets Type 2A (VDDR2A) in a Patient Presenting With Pseudoarthrosis

**DOI:** 10.1155/crie/2434759

**Published:** 2025-04-09

**Authors:** Risa Goldberg, Gunjan Umarji, Serge Jabbour

**Affiliations:** ^1^Department of Internal Medicine, Thomas Jefferson University Hospital, Philadelphia, Pennsylvania, USA; ^2^Division of Endocrinology, Diabetes and Metabolic Disorders, Thomas Jefferson University Hospital, Philadelphia, Pennsylvania, USA

## Abstract

This case report is centered on an atypical presentation of Hereditary Vitamin D-dependent Rickets 2A (VDDR2A), a rare disorder caused by defects in the gene encoding the vitamin D receptor (VDR). While this disorder is primarily autosomal recessive in inheritance, this case demonstrates that a single heterozygous variant in the VDR gene could be linked to both phenotypic and laboratory manifestations of this condition. To understand the pathogenesis of this condition, one must know the typical roles of vitamin D, calcium, and parathyroid hormone (PTH) in maintaining homeostasis in the body. This case report focuses on the underlying pathogenesis of this disorder and demonstrates the variability in the ways this condition can present.

**Summary**

• Heterozygous mutations in the VDR gene may result in clinically significant and symptomatic VDDR2A.
• Physicians should be aware of the significant variability in the way that VDDR2A may present, including both physical manifestations and laboratory abnormalities.
• VDDR2A can be secondary to various mutations in the VDR gene. The clinical picture of this disease continues to evolve as we learn more about the different gene mutations involved in its pathogenesis.
• Once diagnosed, patients with VDDR2A will require calcitriol and calcium supplementation, sometimes requiring extremely high doses of both to achieve a therapeutic effect.

## 1. Introduction

Hereditary Vitamin D-Dependent Rickets Type 2A (VDDR2A) is a disorder characterized by early onset rickets with growth retardation, muscle weakness, alopecia, and teeth abnormalities [1]. These characteristics are secondary to defects in the vitamin D receptor (VDR), causing resistance to the actions of 1,25(OH)_2_ D. This leads to hypocalcemia, secondary hyperparathyroidism, and rickets. These manifestations are seen in the classical presentation of VDDR2A, which traditionally follows an autosomal recessive inheritance pattern. It is proposed that more accurate terminology for VDDR2A could include hereditary 1,25-dihydroxyvitamin D resistant rickets (HVDRR), hereditary resistance to 1,25(OH)_2_D or pseudovitamin D-deficiency type IIa (PDDR IIA) since patients with this disorder may be unable to respond to vitamin D supplementation [1, 2].

This case is unique as our patient was found to have a heterozygous variant in the VDR gene. In this form of VDDR2A, patients usually have no or mild symptoms, and laboratory testing is normal to slightly abnormal [1]. While compound heterozygous variants have been shown to cause clinically significant VDDR2A [3, 4], there are only several case reports in the literature showing symptomatic VDDR2A caused by a single heterozygous VDR variant [5, 6]. These single heterozygous VDR variants were found to exert a dominant negative effect on the wild-type VDR gene. We present here a patient found to be heterozygous for a pathogenic variant in the VDR gene who presented with pseudoarthrosis after spinal surgery, brittle teeth, and hearing loss.

## 2. Case Presentation

A 38-year-old male initially presented to a neurosurgery office with debilitating lower back pain and left leg radiculopathy refractory to treatment with physical therapy and steroid injections. Magnetic Resonance Imaging (MRI) of the spine showed disc bulges at L4-L5 and L5-S1 with foraminal stenoses. He underwent anterior and posterior release and fusion of L4-L5 and L5-S1 with structural allograft placement at L4-S1. After initial resolution, back pain recurred within a year of the procedure. Repeat imaging showed pseudoarthrosis of the L4-5 graft site. At this time, the patient also endorsed having brittle, chipping teeth for many years, and he was referred to an endocrinologist. Upon further prompting in the endocrinology clinic, he endorsed hearing loss in the left ear for 2 years. He did not complain of pain in any bones and joints except in his back. Notably, he endorsed several sports-related fractures as a child, in his left wrist and several ribs. Medical history included anxiety, depression, and peptic ulcer disease. His medications included oxycodone, ibuprofen, pregabalin, mirtazapine, bupropion, and sertraline. He was not on any calcium, vitamin D, or additional supplements. He was unaware of his family history as he was adopted. Social history included active cigaret use (2–4 cigarets daily for 10 years before presentation).

On physical exam, the patient was of normal stature at 1.82 m tall and weighed 89.8 kg with a body mass index (BMI) of 26.8 kg/m^2^. Physical exam was unremarkable except for several broken teeth. He had no evidence of varus or valgus deformity.

**Figure 1:**
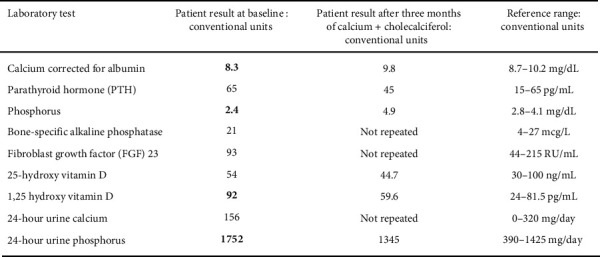
Abnormal values are shown in bold font.

Laboratory studies obtained before he visited with the endocrinologist included the following (Figure 1): corrected calcium 8.3 mg/dL (normal range 8.7–10.2), 25-OH vitamin D 34 ng/mL (normal range 30–100), phosphorus 2.4 mg/dL (normal range 2.8–4.1), intact parathyroid hormone (PTH) 65 pg/mL (normal range 15–65), bone-specific alkaline phosphatase 21 mcg/L (normal range 4–27), 24-h urine calcium 156 mg/day (normal range 0–320), 24-h urine phosphorus 1752 mg/day (normal range 390–1425), FGF-23: 93 RU/mL (normal range 44–215), and 1,25(OH)_2_ D 92 pg/mL (normal range 24–81.5). Prior imaging studies, like plain X-rays of upper/lower limbs, skull, chest, and pelvis, as well as spine MRIs, showed no evidence of rickets. Dual-energy X-ray absorptiometry (DXA) scan showed a lumbar spine Z score of −2.2 and a femoral neck Z score of −1.2.

The high 1,25(OH)2 D and normal FGF-23 ruled out tumor-induced osteomalacia.

Genetic testing through Next Generation Sequencing testing for hypophosphatasia, hypophosphatemic rickets, osteogenesis imperfecta, and other bone disorders showed a single heterozygous variant in the VDR gene, at nucleotide position c.146 + 9dup, consistent with VDDR2A. However, functional and transcriptional activities to see if the heterozygote variant has a dominant-negative effect on wild-type VDR were not performed due to lack of financial support/funding. Thus, we could not confirm with certainty that the above heterozygous variant in the VDR gene was the direct cause of this patient's clinical presentation and VDDR2A. However, the symptoms, signs, and laboratory/imaging findings were all consistent with VDDR2A.

The patient was started on treatment with oral cholecalciferol 50 mcg daily and supplemental calcium 1200 mg orally daily.

Laboratory testing performed after 3 months of treatment showed phosphorus 4.9 mg/dL, corrected calcium 9.8 mg/dL, intact PTH 45 pg/mL, 25-OH vitamin D 44.7 ng/mL, 1,25(OH)_2_ D 59.6 pg/mL, and 24-h urine phosphorus 1345 mg/day. A repeat DXA will be obtained 2 years following the previous one.

## 3. Discussion

This patient's case is atypical in that VDDR2A was diagnosed later in life and found to be possibly related to a heterozygous variant in the VDR gene. VDDR2A is an extremely rare disorder, with case reports describing this condition numbering in the hundreds. There are several variants in the VDR gene on chromosome 12 that have been shown to contribute to VDDR2A, including missense, nonsense, and splice site variants [1]. These lead to the resistance of the VDR to activation by 1,25(OH)_2_D. Children with the autosomal recessive form of VDDR2A may present with symptomatic hypocalcemia in the form of bone pain, muscle weakness, and hypotonia. Delayed growth and fractures may occur. Some children present with alopecia, hypoplasia of the teeth, and dental caries. These features are usually detected in childhood, though there is variability in presentation and symptoms may not become clinically apparent until adulthood [1].

The laboratory abnormalities associated with VDDR2A arise from the inability of active vitamin D to form a complex with the VDR and regulate the target genes needed for calcium and phosphorus homeostasis and bone mineralization. Therefore, this condition is associated with hypocalcemia, low urinary calcium, elevated PTH, hypophosphatemia, hyperphosphaturia, and elevated 1,25(OH)_2_D [1, 2]. These abnormalities are seen in the classical autosomal recessive form of VDDR2A.

Several cases of compound heterozygous variants in the VDR gene leading to the clinical manifestations of VDDR2 have been described [1, 3, 4]. However, only two case reports exist showing a heterozygous variant in the VDR gene leading to this disorder through a dominant negative effect [5, 6]. The first describes a child who presented with early onset rickets, hypocalcemia, secondary hyperparathyroidism, and elevated serum vitamin 1,25(OH)_2_D, who was found to have a heterozygous E420A variant on exon 9 of the VDR gene [5]. The second describes a young boy who was heterozygous for both the Q400LfsX variant and an additional VDR variant, but the Q400LfsX variant was found to have a dominant negative effect on the wild-type VDR gene. The additional variant was maternally inherited and did not affect the wild-type VDR gene. However, both the child and his father were heterozygous for the Q400LfsX variant and presented with rickets during childhood [6].

Our patient's case demonstrates a heterozygous variant at the c.146 + 9dup position in the VDR gene. However, functional and transcriptional activities to see if the heterozygote variant has a dominant-negative effect on wild-type VDR were not performed due to lack of financial support/funding. Thus, we could not confirm with certainty that the above heterozygous variant in the VDR gene was the direct cause of this patient's clinical presentation and VDDR2A. We should also note here that although VDDR2A is transmitted as autosomal recessive and heterozygous carriers being asymptomatic, we cannot rule out the possibility that a heterozygous variant could cause a mild disease. Many other conditions inherited in a recessive fashion could present with mild symptomatology in carriers, such as congenital adrenal hyperplasia, adrenoleukodystrophy, etc. In this case, although the gene testing might not be definitive, the symptoms, signs, and laboratory/imaging findings were all consistent with VDDR2A. His history of fractures as a child, pseudoarthrosis after surgery, chipped teeth, and hearing loss can be attributed to resistance to active vitamin D [1, 2, 7]. His laboratory tests consistent with this disorder included mild hypocalcemia, hypophosphatemia, PTH in the upper limit of normal, and elevated 1,25(OH)_2_D. His history of fractures as a child leads to a significant educational point that children with a history of multiple fractures may warrant further evaluation for metabolic bone disease in the right clinical context.

Treatment for VDDR2A is primarily through calcium and vitamin D supplementation.

Inherent in the name, patients with VDDR2A may be resistant to treatment with vitamin D [2]. However, certain variants have been shown to respond to vitamin D supplementation- this is because certain variants lead to reduced binding affinity of the VDR for active vitamin D, which may be overcome with high doses of supplementation [8]. Patients may also need high doses of calcium supplementation (both orally and intravenously) to obtain treatment response [9].

In summary, our patient presented with symptomatic VDDR2A most likely secondary to a novel heterozygous variant in the VDR gene. His unique presentation of pseudoarthrosis demonstrates the variability in signs and symptoms with which this condition can present.

## Data Availability

The data that support the findings of this study are available from the corresponding author upon reasonable request.

## References

[1] Malloy P., Pike W., Feldman D. (1999). The Vitamin D Receptor and the Syndrome of Hereditary 1,25-Dihydroxyvitamin D-Resistant Rickets. *Endocrine Reviews*.

[2] Feldman D., Malloy P. J. (1990). Hereditary 1,25-Dihydroxyvitamin D Resistant Rickets: Molecular Basis and Implications for the Role of 1,25(OH) 2D3 in Normal Physiology. *Molecular and Cellular Endocrinology*.

[3] Zhou Y., Wang J., Malloy P. J., Dolezel Z., Feldman D. (2009). Compound Heterozygous Mutations in the Vitamin D Receptor in a Patient With Hereditary 1,25-Dihydroxyvitamin D-Resistant Rickets With Alopecia. *Journal of Bone and Mineral Research*.

[4] El Soda S., Madani H. (2011). Compound Heterozygous Mutations in Vitamin D Receptor Gene in Two Sisters With Hereditary Vitamin D Resistant Rickets Type II. *Journal of Endocrinology and Metabolism*.

[5] Malloy P. J., Zhou Y., Wang J., Hiort O., Feldman D. (2011). Hereditary Vitamin D-Resistant Rickets (HVDRR) Owing to a Heterozygous Mutation in the Vitamin D Receptor. *Journal of Bone and Mineral Research*.

[6] Isojima T., Ishizawa M., Yoshimura K. (2015). Hereditary 1,25-Dihydroxyvitamin D-Resistant Rickets (H*VDR*R) Caused by a, VDR, Mutation: A Novel Mechanism of Dominant Inheritance. *Bone Reports*.

[7] Bigman G. (2022). Deficiency in Vitamin D Is Associated With Bilateral Hearing Impairment and Bilateral Sensorineural Hearing Loss in Older Adults. *Nutrition Research*.

[8] Feldman D., Malloy P. J. (2014). Mutations in the Vitamin D Receptor and Hereditary Vitamin D-Resistant Rickets. *BoneKEy Reports*.

[9] Sahay M., Sahay R. (2012). Rickets-Vitamin D Deficiency and Dependency. *Indian Journal of Endocrinology and Metabolism*.

